# Sanguinarine Reverses Pulmonary Vascular Remolding of Hypoxia-Induced PH *via* Survivin/HIF1α-Attenuating Kv Channels

**DOI:** 10.3389/fphar.2021.768513

**Published:** 2021-12-24

**Authors:** Fenling Fan, Yifan Zou, Yousen Wang, Peng Zhang, Xiaoyu Wang, Anthony M. Dart, Yuliang Zou

**Affiliations:** ^1^ Department of Cardiovascular Medicine, The First Hospital of Xi’an Jiaotong University, Xi’an, China; ^2^ School of Economic and Finance, Xi’an Jiaotong University, Xi’an, China; ^3^ Baker Institute, Melbourne, VIC, Australia; ^4^ Department of Cardiovascular Medicine, The Alfred Hospital, Melbourne, VIC, Australia; ^5^ Department of Gynecology and Obstetrics, The First Hospital of Xi’an Jiaotong University, Xi’an, China

**Keywords:** hypoxia-induced pulmonary hypertension, pulmonary vascular remolding, cancer-like mechanism, sanguinarine, survivin, HIF1A, Kv channels

## Abstract

**Background:** Similarities in the biology of pulmonary hypertension and cancer suggest that anticancer therapies, such as sanguinarine, may also be effective in treating pulmonary hypertension. This, along with underlying biochemical pathways, is investigated in this study.

**Methods:** Rats were subjected to 4-week hypoxia (or control) with or without sanguinarine treatment. In addition, pulmonary artery smooth muscle cells (PASMCs) were examined after 24–48 h hypoxia (with normoxic controls) and with or without sanguinirine. Pulmonary artery pressures and plasma survivin levels were measured *in vivo*. *Ex vivo* tissues were examined histologically with appropriate staining. mRNA and protein levels of survivin, HIF-1α, TGFb1, BMPR2, Smad3, P53, and Kv 1.2, 1.5, 2.1 were determined by real-time PCR and Western blot in PASMCs and distal PAs tissue. PASMC proliferation and changes of TGFb1 and pSmad3 induced by sanguinarine were studied using MTT and Western blot. Electrophysiology for Kv functions was measured by patch-clamp experiments.

**Results:** Four-week hypoxia resulted in an increase in serum survivin and HIF-1α, pulmonary artery pressures, and pulmonary vascular remodeling with hypertrophy. These changes were all decreased by treatment with sanguinarine. Hypoxia induced a rise of proliferation in PASMCs which was prevented by sanguinarine treatment. Hypoxic PASMCs had elevated TGFb1, pSmad3, BMPR2, and HIF1α. These increases were all ameliorated by sanguinarine treatment. Hypoxia treatment resulted in reduced expression and function of Kv 1.2, 1.5, 2.1 channels, and these changes were also modulated by sanguinarine.

**Conclusion:** Sanguinarine is effective in modulating hypoxic pulmonary vascular hypertrophy *via* the survivin pathway and Kv channels.

## Background

Pulmonary hypertension (PH) refers to a variety of conditions characterized by elevations in pulmonary arterial pressure. It is a life-threatening disease with about 1% incidence in adults (Nathan et al., 2019). The vascular pathology of PH is characterized by pulmonary vasoconstriction and abnormal (“pseudomalignant”) inward remodeling processes, which may affect all vessel layers (intima, media, and adventitia). A prominent feature virtually in all PH entities is vascular smooth muscle cell (SMC) proliferation, causing medial hypertrophy of the intra-acinar muscular resistance vessels and muscularization of the normally nonmuscularized precapillary arterioles. Overall, these structural changes suggest a switch from “quiescent” toward “pro-proliferative,” “apoptosis-resistant,” and “pro-inflammatory” vascular cell phenotypes. It has become increasingly clear that PH can be viewed as a proliferative disease and has an incredible number of pathogenic mechanisms similar to cancer. The vast majority of cancer hallmarks during disease progression (except tissue invasion and metastasis) are also shared by pulmonary vascular cells in PH patients (Pullamsetti et al., 2020). Recent experimental and conceptual advances (Pullamsetti et al., 2020) in the cancer cell metabolism, evading immune destruction and inflammation by innate immune cells, provide the field of PH with the unique opportunity to target the metabolism and immune/inflammation axis.

There are several underlying causes and five categories are recognized. These are pulmonary arterial hypertension (PAH, Type 1), pulmonary hypertension due to left heart or lung/hypoxia diseases (Type 2 and Type 3, respectively), PH due to pulmonary arterial stenosis (Type 4), and PH due to miscellaneous cause (Type 5). The various types of PH differ widely with respect to their prevalence and treatment. Two most frequent causes of pulmonary hypertension are left heart diseases and hypoxic lung diseases. Global studies of chronic obstructive lung disease (COPD) stage IV showed that up to 90% have mPAP >20 mmHg, with most ranging between 20 and 35 mmHg. Approximately 1–5% of COPD patients have mPAP >35–40 mmHg at rest (Nathan et al., 2019). Whereas, the prognosis of fibrotic idiopathic pulmonary disease (IIP) with PH is even worse than that of idiopathic pulmonary arterial hypertension (IPAH) (Nathan et al., 2019). Unfortunately, none of the drugs licensed for treatment of PAH have proven any survival effect in patients with pulmonary hypertension due to left heart disease or lung/hypoxia disease. One possible important reason is that current therapies have focused on vasodilators, which fail to reverse vascular remodeling, a key outcome for most types of PH (Thenappan et al., 2018). Based on many points of overlap for most PH, including deregulated inflammation, sustained proliferation, and escape from apoptosis, vascular remolding of PH has emerged as cancer-like, as previously mentioned (Pullamsetti et al., 2020), providing a possibility that anticancer reagents would be a therapeutic perspective for targeting vascular remolding of PH (Thenappan et al., 2018).

Sanguinarine, a benzophenanthridine alkaloid derived primarily from the bloodroot plant, has antioxidant, anti-inflammatory, proapoptotic, and growth inhibitory effects on different tumor cells (Fu et al., 2018). Survivin has been believed to be the strongest inhibiting protein of apoptosis and is widely involved in various cancers (Li et al., 2019a; Tian et al., 2018). The transcription factor hypoxia inducible factor-1alpha (HIF-1α) mediates adaptive responses to oxidant stress, promotes inflammatory gene expression, and contributes to tumor proliferation (Tian et al., 2018; Luo et al., 2019; Cheng et al., 2019; Diez-Calzadilla et al., 2021). Together, both survivin and HIF-1α are involved in inflammation, oxidative stress, proliferation, and apoptosis imbalance which are all key pathways for pulmonary vascular remolding in PAH (Thenappan et al., 2018; Pullamsetti et al., 2020; Diez-Calzadilla et al., 2021). Furthermore, oxygen-sensitive voltage-dependent K⁺ (Kv) channels regulate cell proliferation and apoptosis both in PH and tumors (Hasan and Jaggar, 2018; Haworth and Brackenbury, 2019). The similarity in disordered molecular pathways between cancer and pulmonary hypertension suggests the possibility that therapy which is successful in cancer may also be effective in pulmonary hypertension. Therefore, in this study, the plant-originating sanguinarine, a novel inhibitor of survivin, was investigated for reducing pulmonary vascular remolding. We hypothesized that the mechanism of reversing pulmonary vascular remodeling involved upregulation of Kv channels via survivin/HIF-1α in pulmonary artery smooth muscle cells (PASMCs). The mechanism would further illustrate the similarity of cancer and PAH. The results would add important data for revealing a new pathway of reversing pulmonary vascular remolding likely leading to a better long-term outcome.

## Materials and Methods

### Animals

The experimental protocols in this study were approved by the Laboratory Animal Administration Committee at Xi’an Jiaotong University and performed according to the Guide for the Care and Use of Laboratory Animals, published by the US National Institutes of Health. Male Sprague–Dawley rats (weight 180−250 g, 6–8 weeks old) were purchased from the Laboratory Animal Center at Xi’an Jiaotong University (Xi’an, China). The rats were randomized into control and hypoxia groups (*n* = 28, respectively). Hypoxic rats were housed in a hypoxic (10% O_2_) Plexiglas cabin for 8 h/day for 4 weeks (Luo et al., 2014), whilst controls were maintained under normoxic conditions. Half of the rats in each group were given 30 mg/kg/day of sanguinarine (Macklin Inc., Shanghai, China) by gavage (Control + Sang, Hypoxia + Sang, respectively). The remaining half of control and hypoxia groups were provided with an equal volume of 5 ml/kg/day of saline.

### Hemodynamics Measurement

After 4 weeks, the rats were anesthetized with 100 g/L of chloral hydrate (3 ml/kg) intraperitoneally and placed on an operating table. Subsequently, a percutaneous jugular vein-catheter, through the right atrium and ventricular, was installed into the main pulmonary artery and then connected to a pre-corrected zero value pressure transducer of a multiple electroconductive physiological recorder (Shanghai Yuyan SciTech Instruments Ltd., China) to record the pulmonary artery pressure (including systolic, diastolic, and mean pressures, sPAP, dPAP, and mPAP) with the heart rate.

### Assessment of Right Ventricle Hypertrophy and Pulmonary Artery Remodeling

After the rats were sacrificed, the heart, lung, and liver were immediately isolated and weighed. The right ventricle (RV) was dissected from the left ventricle and septum (LV + S), and the Fulton index [RV wet weight/(LV + S)] was calculated as an index of right ventricular hypertrophy (RVHI). Then, the right lower lobes of the lung tissues were collected and fixed with 4% paraformaldehyde, dehydrated, paraffin-embedded, cut into 10 μm sections, stained with hematoxylin and eosin (HE), and observed for pulmonary arteriole morphology under a light microscope (Olympus, Japan). 3 HE–stained lung tissue sections, each with at least three pulmonary arterioles, were randomly selected from every group for pulmonary arteriole measurement. Three views for each arteriole was observed, and the outer and inner circumference and wall thickness were calculated using Image-Pro Plus 6.0 software. The average values were used to assess vascular proliferation.

### Enzyme-Linked Immunosorbent Assay

The content of survivin and HIF-1α in rat serum was determined by the ELISA method, using the Rat Survivin (Thermo Scientific, United States) and HIF1α (Abbkine, United States) ELISA kits following the manufacturer’s instructions. In brief, 20 μL of each standard, control, and samples (serum) in duplicate was dispensed with new disposable tips into appropriate wells on a microplate coated with survivin and HIF1α antibodies. After that, each microplate well was filled with 200 μL enzyme conjugate, thoroughly mixed, and then incubated for 60 min at room temperature. After incubation, the solution in the wells was briskly shaken out and rinsed out four times with 350 μL diluted washing solution per well. Furthermore, 100 μL substrate solution was added to each well of the microplate, which was then re-incubated for 15–20 min at room temperature. After that, the enzymatic reaction was stopped by adding 100 μL stop solution to each well. Finally, absorbance was determined by using an ELISA reader (xMark™ Microplate Absorbance Spectrophotometer, Bio-Rad Laboratories Inc.) at 450 nm for quantification of protein abundances.

### Preservation of Tissue Specimens

Second- to third-degree branches of the pulmonary artery were isolated from the lobes of the lungs, cleaned off blood in cold phosphate-buffered saline, dissected and removed the endothelium, frozen in liquid nitrogen, and stored at −80°C for subsequent mRNA or protein extraction.

### Preparation and Treatment of Isolated Pulmonary Artery Myocytes

To functionally characterize Kv channels by whole-cell patch-clamp, PASMCs were freshly dissociated from intralobar pulmonary arteries of rats from control, Control + Sang, Hypoxia, and Hypoxia + Sang groups, as described previously (Fan et al., 2019). In brief, following initial PA equilibration in hypoxic iced low-Ca^2+^ Krebs solution, small pieces of muscle layers were stood for 20 min at room temperature prior to digestion with 4 mg/ml papain, 1.25 mg/ml BSA, and 2 mg/ml DTT in 2 ml hypoxic low-Ca^2+^ dissolution solution. Following threefold washing in low-Ca^2+^ solution, separated PASMCs were stored in hypoxic low-Ca^2+^ solution with 0.5% BSA at 4°C for use within 3–4 h.

PASMCs for the remaining cell experiments were isolated from normal adult SD rats and identified by immunohistochemical staining with α-SMC. Rat PASMCs were exposed to hypoxia (1% O_2_, 5% CO_2_, and rest N2) in the 1% (v/v) FCS and 1% (m/v) penicillin and streptomycin M199 medium for the indicated period of time. All other measurements were performed under normoxic conditions, starting 30 min after termination of hypoxia.

For cells in the treatment groups, a series of doses of sanguinarine (0.05, 0.5, 5, and 50 μM; Macklin Inc., Shanghai, China) was separately added either in the hypoxia or normoxia medium for the indicated period. 1-10-100 nmol/L survivin selective inhibitor YM155 [YM155 (Sepantronium bromide) AbMole, United States.] was used as a positive control versus DMSO as a negative control. Cells were harvested at 24 h for 24-h MTT experiments, and the rest were collected at 48 h for 48-h MTT and RNA and protein extraction.

### Immunofluorescence Staining

Isolated cells grown on glass slides were fixed with 4% formaldehyde in PBS for 30 min and incubated in blocking solution for 30 min (PBS containing 2% BSA-0.1% Triton X-100). Cells were incubated with primary antibodies against α-SMA, F-actin, collagen I, and collagen III for 1 h and incubated for 30 min at 37°C with secondary antibodies (Jackson ImmunoResearch). The cell nuclei were stained with 4′,6′-diamidino-2-phenylindole (DAPI; Sigma-Aldrich). Cells were observed with fluorescence microscopy (Olympus).

### MTT

PASMC proliferation was determined by a MTT method. In brief, at the end of treatments, 20 mg of 5 mg/ml MTT solution was added to each well, and PASMCs were incubated for 24 and 48 h at 37°C. Then, the culture medium was removed, and 150 l DMSO was added to each well and incubated for 10 min to solubilize the formazan salt crystals. The formazan within cells was quantified at 490 nm using an enzyme-linked immunosorbent assay reader (Biotek, ELK 800, United States). Each group had six wells, and the average optical density value of those six wells was used as the result of each experiment.

### Real-Time PCR

Total RNA was extracted using a TRIzol reagent (Invitrogen, United States), according to the manufacturer’s instructions. The concentration and quality of isolated RNAs were determined using an ultraviolet spectrophotometer and by electrophoresis on agarose gel, respectively. The cDNA was synthesized using a Reverse Transcriptase kit (Takara, Japan). The specific primer sequences used for the real-time PCR (Rt-PCR) are presented in Table 1. The Rt-PCR was performed with the SYBR Premix Ex Taq (Takara, Japan) on the iQ5 Multicolor RT-PCR Detection System (Bio-Rad, CA, United States). The relative expression of genes was calculated from cycle thresholds (Ct) and normalized to β-actin using the 2^**-**ΔΔ**Ct**^ (Livak) method.

**TABLE 1:** Primer sequences for the Real-time PCR in the study.

Primer	Forward/reverse (5′→3′)	Primer sequence	Product size(bp)
Kv1.5	Forward	CGGGTGTTCCGCATCTTC	169
	Reverse	TTC​CCT​GGT​TGT​CAG​CCT​CT	
Kv2.1	Forward	CCA​AAA​GTC​TCC​ACG​GGA​GT	170
	Reverse	GCA​TTT​CTC​TTG​AGC​CCC​AG	
Kv1.2	Forward	TGG​GCA​CCC​TCA​AGA​CAC​CTA​T	271
	Reverse	TAA​GGG​CAC​ATT​CAC​AGG​TCG​C	
HIF-1α	Forward	GAA​ACT​TCT​GGA​TGC​TGG​TG	167
	Reverse	CAA​ACT​GAG​TTA​ATC​CCA​TG	
p53	Forward	CGA​GCA​CTG​CCC​AAC​AAC​AC	224
	Reverse	TGG​CGG​GAG​GTA​GAC​TGA​CC	
Survivin	Forward	TCT​CAA​GGA​CCA​CCG​CAT​CT	239
	Reverse	CGC​ACT​TTC​TCC​GCA​GTT​TC	
TGFb1	Forward	GCA​ACA​ACG​CAA​TCT​ATG​AC	301
	Reverse	CCC​TCT​ATT​CCG​TCT​CCT​T	
SMAD3	Forward	GGGCTTT GAGGC TGTCTA	224
	Reverse	CCCTTTACT CCCA GTGTCT	
GAPDH	Forward	CAC​TGT​GCC​CAT​CTA​CGA​GG	155
	Reverse	TAA​TGT​CAC​GCA​CGA​TTT​CC	

### Western Blot

Total protein was extracted using RIPA buffer, and the concentration was quantified using a BCA Protein Assay Kit (Genshare Biological, China). Equal amounts of protein were loaded onto 10% SDS-PAGE gels for electrophoresis, transferred onto the PVDF membrane, blocked by 5% skim milk, and incubated with the primary antibody overnight at 4°C, including anti-survivin (1:500; Abcam, United States), anti-TGFb1(1:100; Abcam, United States), anti-pSmad3 (1:1,500; Abcam, United States), anti-BMPR2 (1:1,500; Abcam, United States), anti-HIF1α(1:500; Abcam, United States), anti-P53 (1:1,000; Abcam, United States), anti-Kv1.2 (1:400; Sigma, United States), anti-Kv1.5 (1:200; Abcam, United States), anti-Kv2.1 (1:500; Sigma), and anti-GAPDH (1:1,000; Epitomics, United States). The membrane was washed and incubated with HRP-conjugated goat anti-rabbit IgG (1:4,000; Abcam) at room temperature for 1 h. The blots were visualized using the chemiluminescence system and quantized by Quantity One.

### Patch-Clamp Electrophysiology

Cells for patch-clamp experiments were performed under hypoxic or normoxic conditions as indicated. Drug interventions were as indicated in the “Results” section. The record of K+ current (IK) and EM in the PASMCs was performed using an Axopatch 200B patch-clamp amplifier and 3- to 5-M micropipettes. PASMCs were voltage-clamped at −70 mV, and currents were evoked by steps of 200-ms duration from −70 to +70 mV. Membrane currents were filtered at 5 kHz, digitized using a Digidata 1320A interface (Axon Instruments, Foster City, CA, United States), and analyzed using pCLAMP software.

### Statistical Analysis

Values are given as mean ± SEM (standard error of the mean) or SD (standard deviation) or percentage (N). Calculations were performed using the GraphPad Prism software package (version 4.0.1; GraphPad Software, Inc., La Jolla, CA, United States). Statistical tests were two-sided, and data were tested for normality. Comparisons between two groups were assessed with a student “t” test. The “t” test was used with a Bonferroni correction for multiple comparisons too. The χ^2^ test was used to evaluate the differences among groups with ordinal data. Dose–response was analyzed by a four-parameter logistic regression model. A *p*-value of less than 0.05 was considered significant.

## Results

### Effects of Sanguinarine on Pulmonary Artery Pressures and Levels of Serum Survivin and HIF1α in Chronic Hypoxic Rats

**FIGURE 1:**
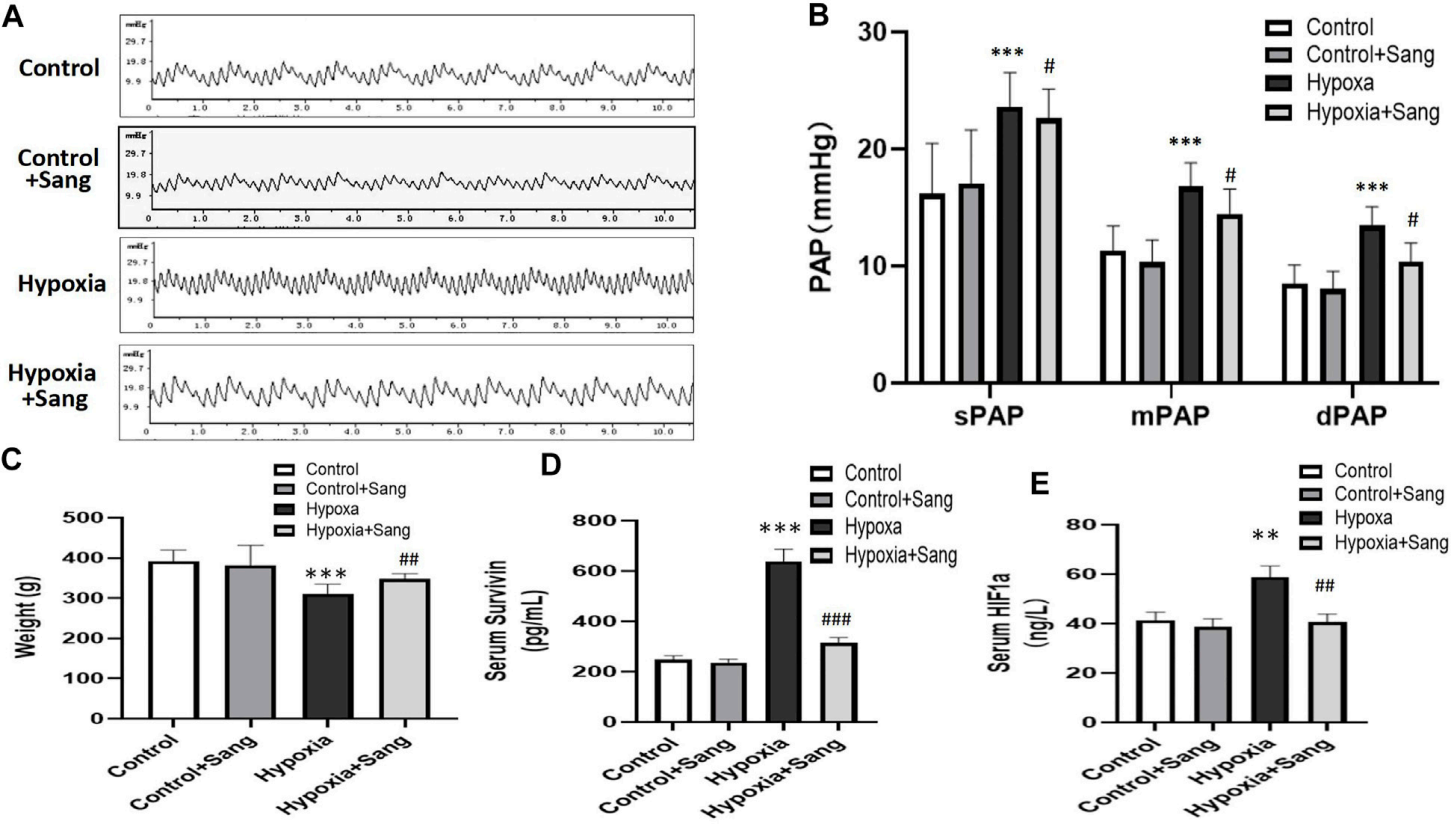
Body weight, pulmonary artery pressures, and serum survivin and HIF1α of the rats: representative traces of pulmonary artery pressure (PAP) **(A)** and the value of systolic pressure (sPAP), mean pressure (mPAP), and diastolic pressure (dPAP) **(B)**, and body weight of the rats **(C)**. Survivin and HIF1α levels in serum **(D,E)** in control (*n* = 12), control treated with sanguinarine (*n* = 12) and untreated hypoxia PH rats (*n* = 12), and 28 days after sanguinarine treatment (*n* = 12). Survivin and HIF1α were measured by ELISA. Results are mean ± SEM. **p<0.01,***p<0.001 Hypoxia PH vs control group. ##*p*<0.01 Hypoxia PH vs Hypoxia PH + Sanguinarine group.

There was no significant difference among the groups at baseline in body weight. After 4 weeks, weight gain was less in hypoxic that control rates. However, sanguinarine treatment significantly improved the nutritional status (Table 2; Figure 1C). Meanwhile along with increase of pulmonary artery pressure (PAP) (including systolic, diastolic, and mean pressures, sPAP, dPAP, and mPAP), in rats exposed to hypoxia, the levels of serum survivin and HIF1α were elevated, compared with those in the control animals. However, PAP and serum survivin and HIF1α remained at lower levels in hypoxic rats treated with sanguinarine than those in untreated hypoxic rats (Table 2; Figures 1A,B,D,E).

**TABLE 2:** Groups and *In vitro* indexes of study rats.

Parameter	Control	Control + Sang	Hypoxia	Hypoxia + Sang
Survival(%), (n/N)	100 (24/24)	100 (24/24)	75 (18/24)**	87.5 (21/24) ##
Body weight(g)				
Week 0	229.82 ± 5.82	231.46 ± 8.65	230.80 ± 7.31	229.96 ± 9.41
Week 4	391.9 ± 28.11	381.5 ± 50.02	310.4 ± 24.71*	347.8 ± 13.40 #
Pulmonary Pressure(mmHg)				
sPAP	16.19 ± 4.3	17.04 ± 4.6	23.65 ± 2.93***	22.64 ± 2.53#
mPAP	11.33 ± 2.11	10.33 ± 1.90	16.81 ± 2.04	14.48 ± 2.11 #
dPAP	8.48 ± 1.62	8.05 ± 1.54	13.47 ± 1.62	10.38 ± 1.64 #
Serum survivin (pg/ml)	248.16 ± 68.8	236.37 ± 63.2	639.32 ± 106.48***	314.66 ± 71.28 ###
Serum HIF1a (ng/L)	41.36 ± 6.74	38.69 ± 7.18	58.84 ± 8.32**	40.65 ± 5.87##
RVHI	0.275 ± 0.023	0.269 ± 0.030	0.385 ± 0.130***	0.283 ± 0.080 ###
Inner circumferencce (mm)	9.61 ± 2.30	9.22 ± 1.91	5.20 ± 1.62***	7.84 ± 2.38 #
Thickness (mm)	1.30 ± 0.42	1.25 ± 0.52	2.82 ± 0.64***	1.92 ± 0.44 #

*p<0.05; **p < 0.01;***p < 0.001 Hypoxia vs. control. #*p* < 0.05 ##*p* < 0.01;###*p* < 0.001 Hypoxia + Sang vs. Hypoxia.

### Effects of Sanguinarine on Remodeling of Pulmonary Arteries and Right Heart as Well as the Survival Rate in Chronically Hypoxic Rats

**FIGURE 2:**
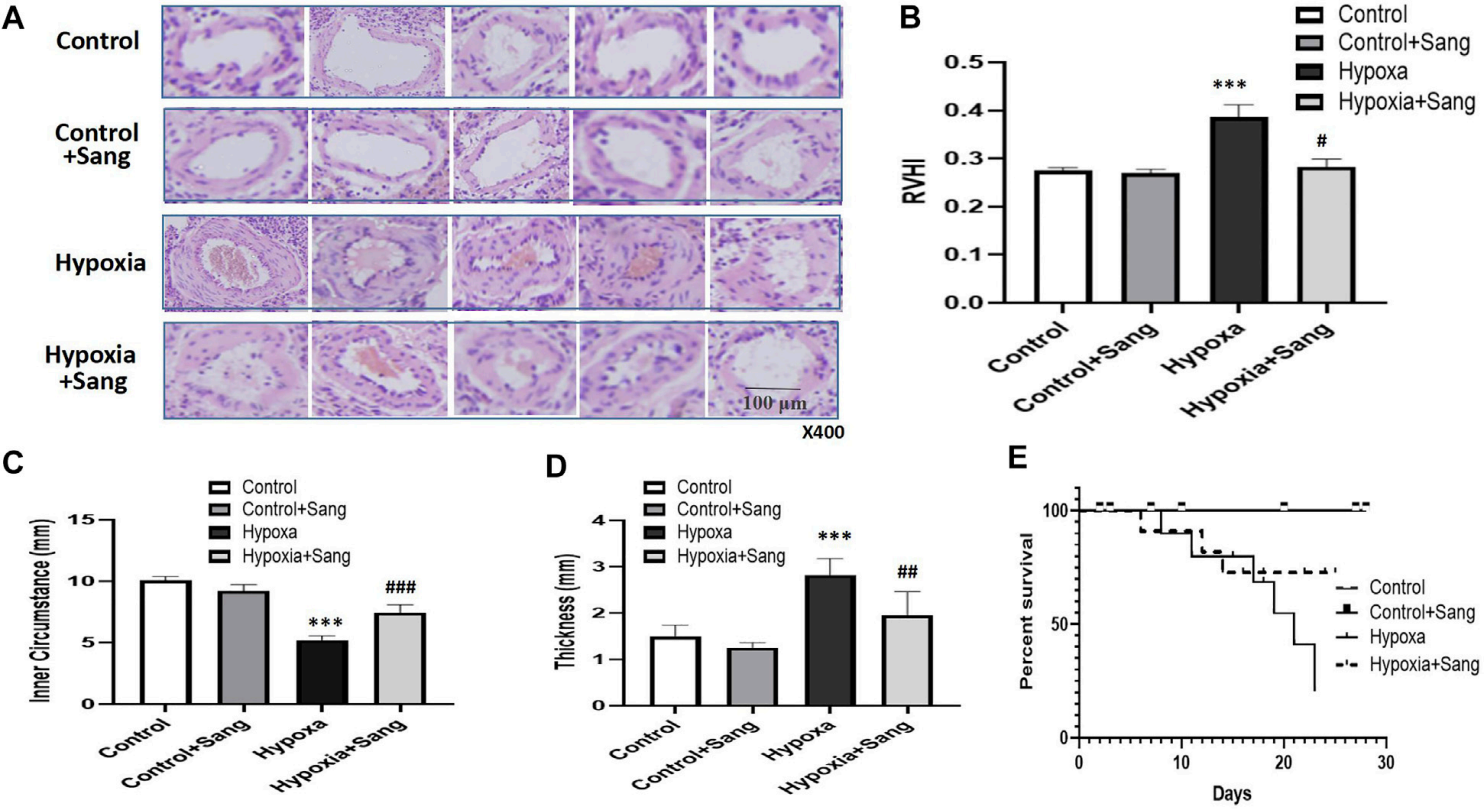
Histology **(A)**, HE staining (under 400× magnification) of pulmonary arteries, and morphometric measures **(B–D)** in control rats (*n* = 12), control treated with Sanguinarine (*n* = 12) and hypoxia PH rats before (*n* = 12) and after (*n* = 12) 28 days of sanguinarine treatment. Survival rate curve are shown in figure 2E. RVHI was calculated as (RV weight/LV + S weight). Results are mean ± SEM. ***p<0.001 Hypoxia PH vs control group. ##*p*<0.01, ###*p*<0.001, Hypoxia PH vs Hypoxia PH + Sanguinarine group.

Histological examination showed that the right ventricles were enlarged, the lungs and livers looked edematous and congested, and pleural effusion and ascites were visible in rats subjected to hypoxia when rats were sacrificed after 4 weeks. All the aforementioned signs were significantly less after sanguinarine treatment (data not shown here). Furthermore, rats exposed to hypoxia showed hypertrophy of pulmonary arteries with a thicker medial smooth muscle layer and smaller inner lumen area. All these indices indicative of remodeling of the pulmonary arteries and right heart were reduced dramatically in the Sang + Hypoxia group (Table 2, Figures 2A,B,C,D). Sanguinarine also improved survival in rats exposed to hypoxia (Figure 2E).

### Effects of Sanguinarine on Proliferation and Fibrosis of Smooth Muscles of Pulmonary Arteries

**FIGURE 3:**
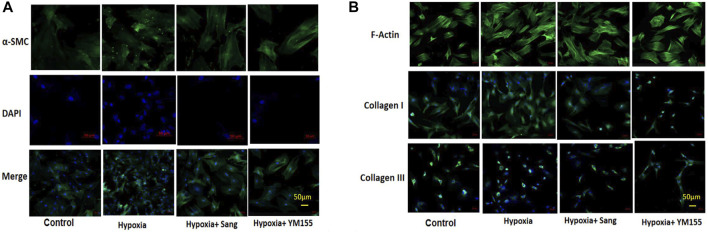
Immunofluorescence staining of PASMC proliferation and fibrosis. α-SMC staining for PASMC, DAPI for nucleus, and merge for both cytoplasm and nucleus staining. **(A)** F-actin, collagen I, and collagen III represent the fibrosis in PASMCs **(B)**.

**FIGURE 4:**
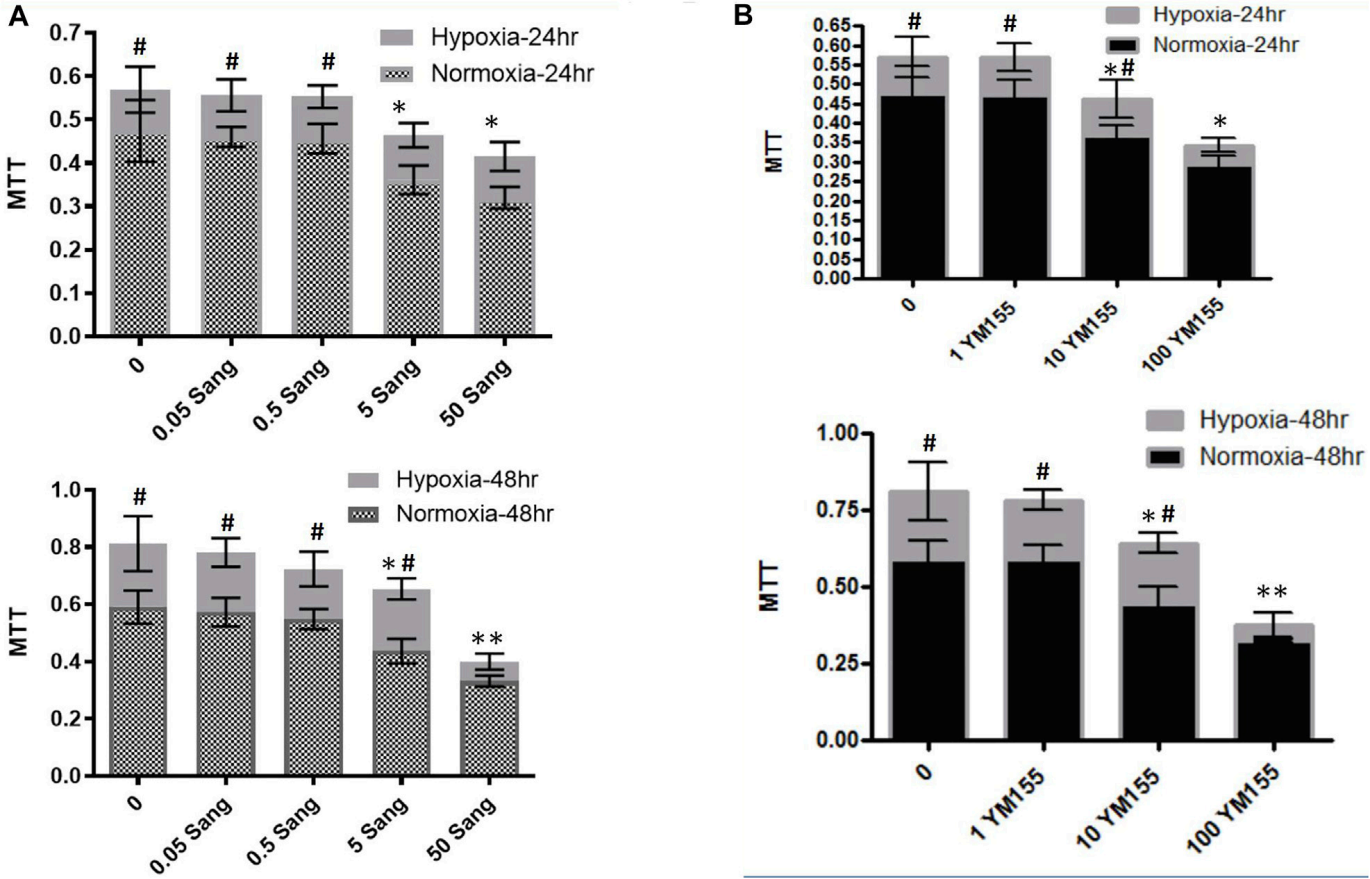
MTT experiments in PASMCs for cell proliferation. MTT experiments for PASMC proliferation under treatment of different sanguinarine concentrations **(A)**. The cell numbers in the hypoxia group during sanguinarine treatment was presented with gray bar, while ones in the normoxia group during sanguinarine treatment were with combination of gray and small square **(A)**. Survivin selective inhibitor YM155 was used as a positive control. MTT experiments for PASMC proliferation under treatment of different YM155 concentrations **(B)**. The same style of symbols as in **(A)**. It means that cell numbers in the hypoxia group during YM155 treatment were presented with light gray bar, while ones in the normoxia group during YM155 treatment were with black bar **(B)**. *p < 0.05,**p < 0.01, Hypoxia PASMC with different doses of Ssnguinarine or YM155 vs control PASMC without treatment. #*p* < 0.05,##*p*<0.01 Hypoxia PASMC vs normoxia PASMC.

In the cell experiments, the PASMCs and the proliferation were identified by immunofluorescence staining with α-SMC (Figure 3) and then determined with the MTT method (Figure 4). Both sanguinarine and survivin selective inhibitor YM155 significantly inhibited hypoxia-induced PASMC proliferation (Figures 3A, 4), accompanied by decreases in SMC F-actin cytoskeleton, collagen I, and collagen III (Figure3B). These results are consistent with notion that sanguinarine prevents or inhibits pulmonary artery remodeling via the survivin pathway.

### Effects of Sanguinarine on HIF1α–Survivin/TGFb/Smad Pathways

**FIGURE 5:**
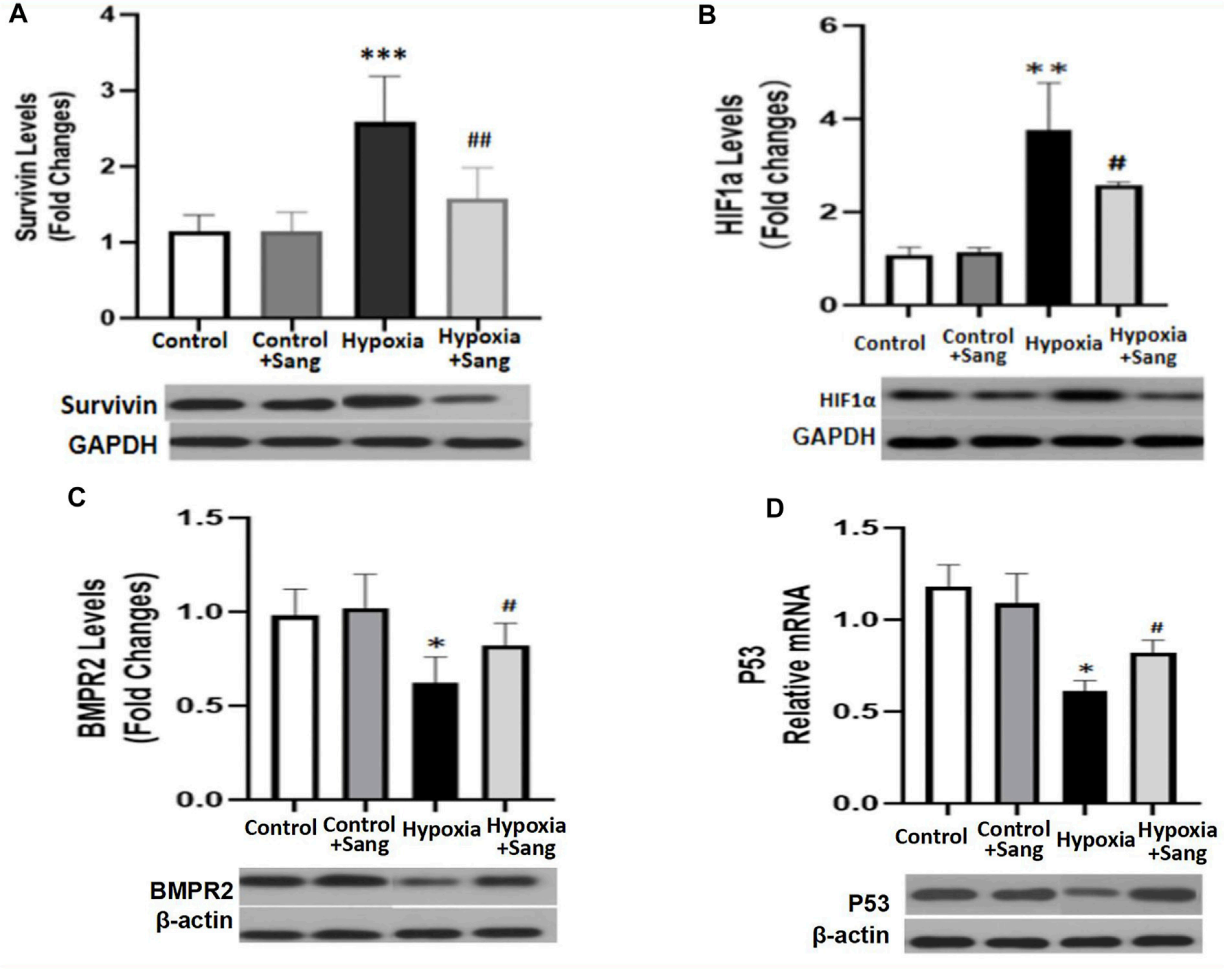
mRNA and protein expressions of Survivin/HIF1α **(A,B)** and BMPR2/p53 **(C,D)** in distal PA tissue from different rat groups: control (*n* = 12) and hypoxia PH rats without (*n* = 24) and following (*n* = 12) sanguinarine treatment. The upper panel shows relative mRNA expression. The lower panel shows Western blots of protein expression. A different gel was used for each receptor, and the respective GAPDH band is shown for each. Gels were analyzed with a Biorad Universal Hood II Molecular Imager Gel System. Results are mean ± SEM. ***p < 0.001 **p < 0.01 *p < 0.05 Hypoxia PH vs control group. ##*p* < 0.01 #*p* < 0.05 Hypoxia PH vs Hypoxia PH + Sanguinarine group.

**FIGURE 6:**
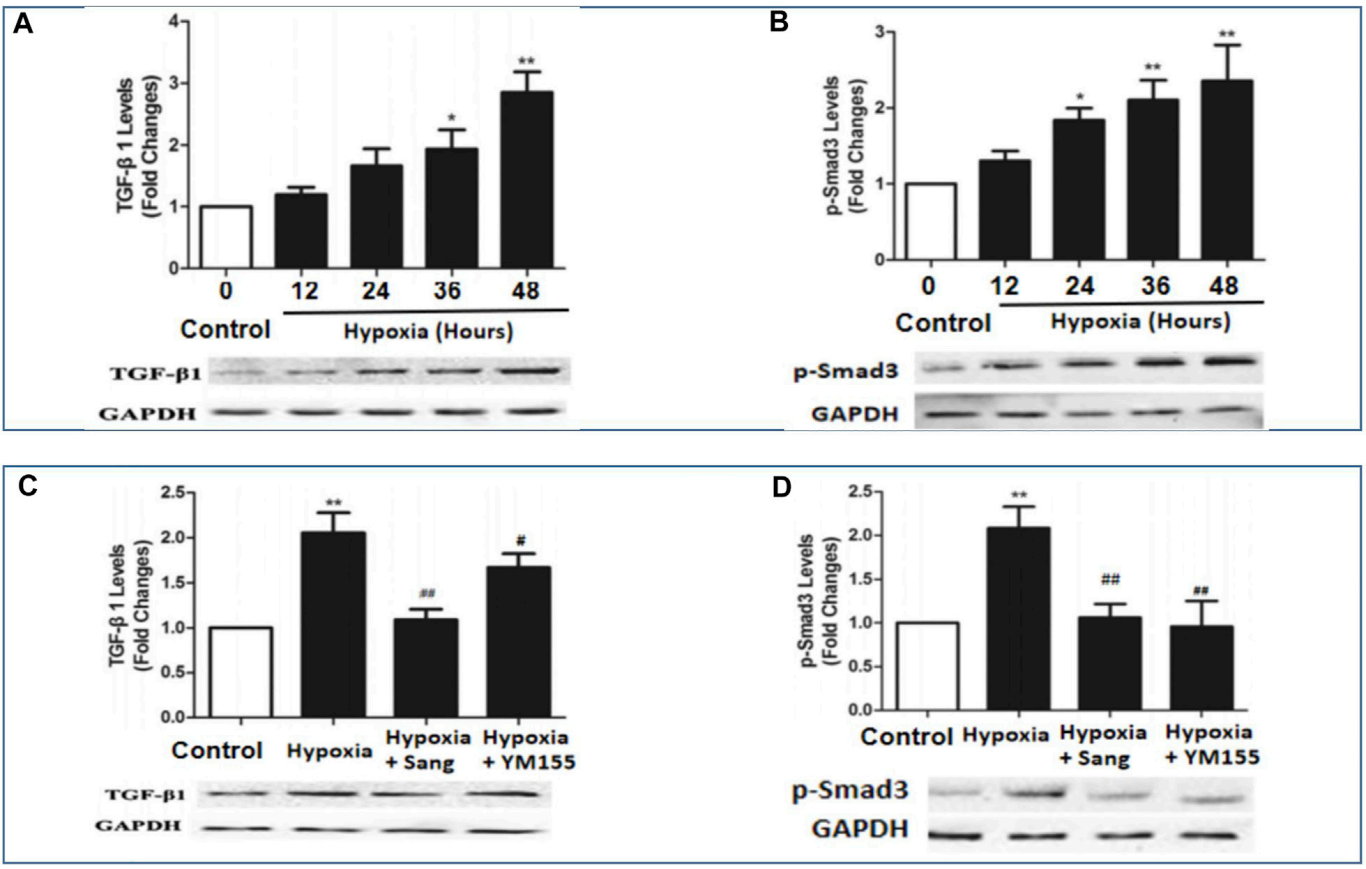
mRNA and protein expressions of BMPR2 and pSmad3 in hypoxia-exposed PASMC **(A,B)** and PA from different rat groups **(C,D)** including control (*n* = 12) and hypoxia PH rats without (*n* = 12) and following (*n* = 12) sanguinarine treatment. Pulmonary artery tissues were obtained from the third and subsequent bifurcations. YM155 was used as a positive control. The upper bars show relative mRNA expression of PASMC under different hours of hypoxia exposure or PA tissue from different rat groups. The lower bands show Western blots of protein expression. A different gel was used for each receptor, and the respective GAPDH band is shown for each. Target gene bands compared with housekeeper GAPDH were analyzed with a Biorad Universal Hood II Molecular Imager Gel System. Results are mean ± SEM. *p < 0.05,**p < 0.01, Hypoxia PH vs control group. #*p* < 0.05,##*p*<0.01 Hypoxia PH vs Hypoxia PH + Sanguinarine group or Hypoxia PH vs Hypoxia PH + YM155 group.

HIF1α is one of the critical mediators in hypoxia-induced injury either by regulating proliferation and apoptosis, oxygen stress–related genes such as SMAD and survivin or interacting with other transcription factors such as p53. Thus, inhibiting the HIF1α pathway has been believed to be a potential therapy for cancer, as well as for hypoxia-induced diseases (Su et al., 2019; Gaber et al., 2021). Accordingly, in this study, we examined whether the inhibiting effects of sanguinarine on hypoxia-induced PASMC proliferation and fibrosis were mediated by the HIF1α/TGFβ/Smad pathway or/and by its feed-forward loop with survivin. The results showed that both serums HIF1α and survivin were significantly increased in rats after 4-week hypoxic exposure (Figures 1D,E, Hypoxia vs. Control, P<0.01), but restored if rats were treated with sanguinarine during this period (Hypoxia + Sang v.s. Hypoxia, P<0.01) (Table 2; Figures 1D,E). Furthermore, their gene expressions of mRNA and protein changed in the same ways with above serum levels of HIF1α and survivin (Figures 5A,B). Meanwhile, it was detected that both mRNA and protein expressions of TGFβ1 and pSmad3 were increased in a time-dependent manner in hypoxic PASMCs *in vivo* (Figures 6A,B). Additionally, TGFβ1 and pSmad3 gene expressions were dramatically decreased after sanguinarine intervention (Figures 6C,D) *in vitro* as well. These effects were similar with the positive control reagent, survivin selective inhibitor YM155 (Figures 6C,D). These results imply that HIF-1α/TGFβ/Smad and survivin are importantly involved in the hypoxia-induced PH mechanism so that sanguinarine being with the survivin inhibitor reversed hypoxic-related pulmonary remolding.

## Effects of Sanguinarine on BMPR2/Smad3/p53 Pathways

Bone morphogenetic protein type 2 receptor (BMPR2)/BMP signaling is a typical molecular mechanism for not only primary but also hypoxic PH. It was proposed TGFβ, working partially via its downstream Smad, is critical in lung vascular remodeling (Takahashi et al., 2006; Liu et al., 2013). In this study, the changes of BMPR2, pSmad, and p53 were examined (pSmad results presented in result part 4) (Figures 6B,D). Hypoxia increased the mRNA and protein of BMPR2 and p53 (Hypoxia group vs. Control, *p* < 0.05), but these effects were reduced by sanguinarine treatment (Sang + Hypoxia group vs. Hypoxia group, *p* < 0.05) (Figures 5C,D). These results confirmed the important role of BMPR2/Smad3/p53 in hypoxia PH.

## Effects of Sanguinarine on O_2_-Sensitive Kv Channels

**FIGURE 7:**
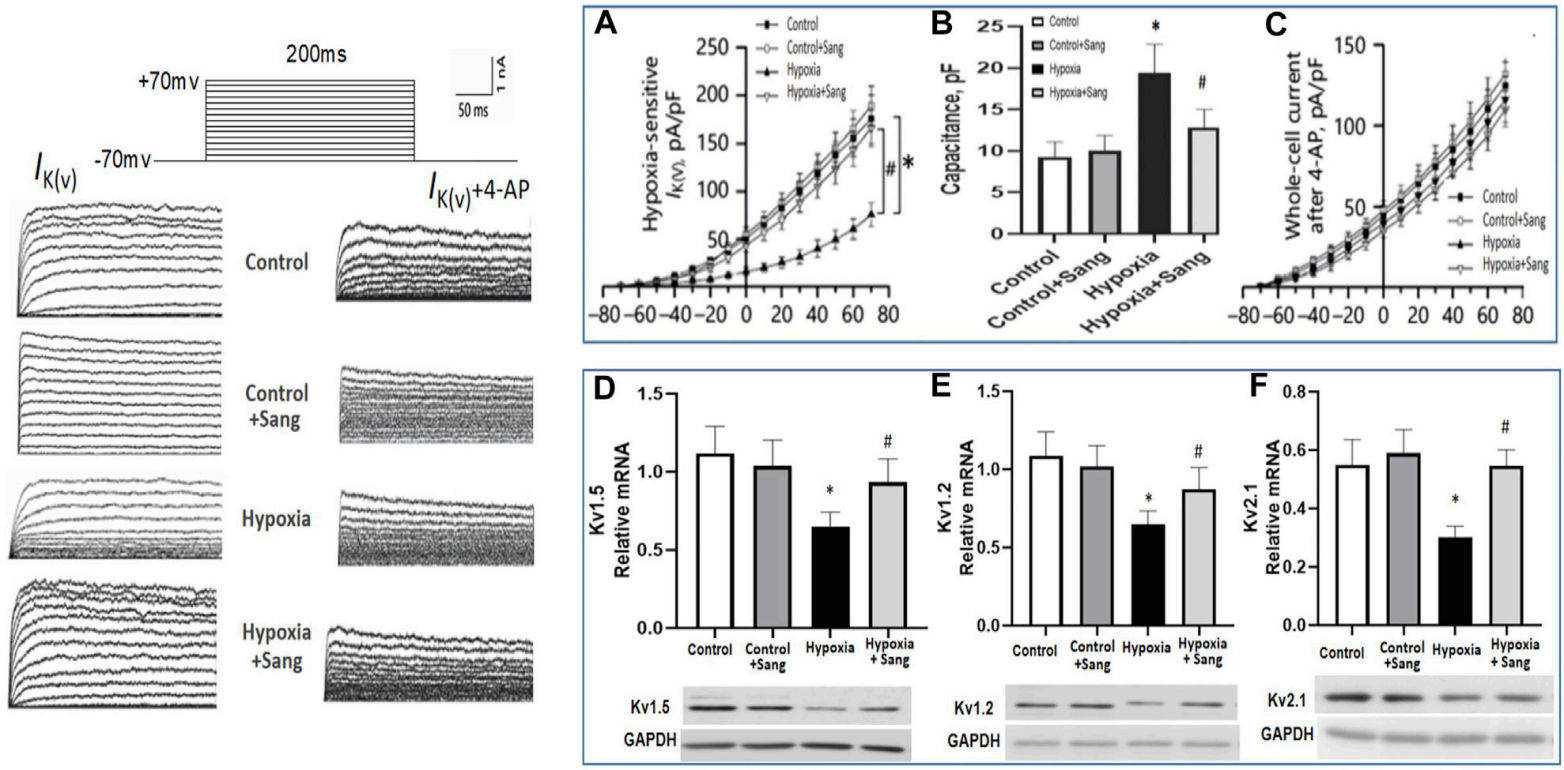
Whole-cell K+ current (IK) and EM of PASMCs were measured by whole-cell patch-clamp in normoxia (control) and hypoxia setting with or without treatment as shown. **(A)**: In the absence of the Kv channel blocker 4-AP. **(B)**: Capacitance was measured in PASMC with different treatment as shown. **(C)**: In the existence of 4-AP. **(D–F)**: RT-PCR and Western blots of Kv channels in rats pulmonary arteries from different groups: control (*n* = 12) and hypoxia PH rats without (*n* = 12) and following (*n* = 12) sanguinarine treatment. The upper panel shows relative mRNA expression. The lower panel shows Western blots of protein expression. A different gel was used for each gene, and the respective GAPDH band is shown for each. Gels were analyzed with a Biorad Universal Hood II Molecular Imager Gel System. Results are mean ± SEM. *p<0.05, Hypoxia PH vs control group. #p < 0.05 Hypoxia PH vs Hypoxia PH + Sanguinarine group.

**FIGURE 8:**
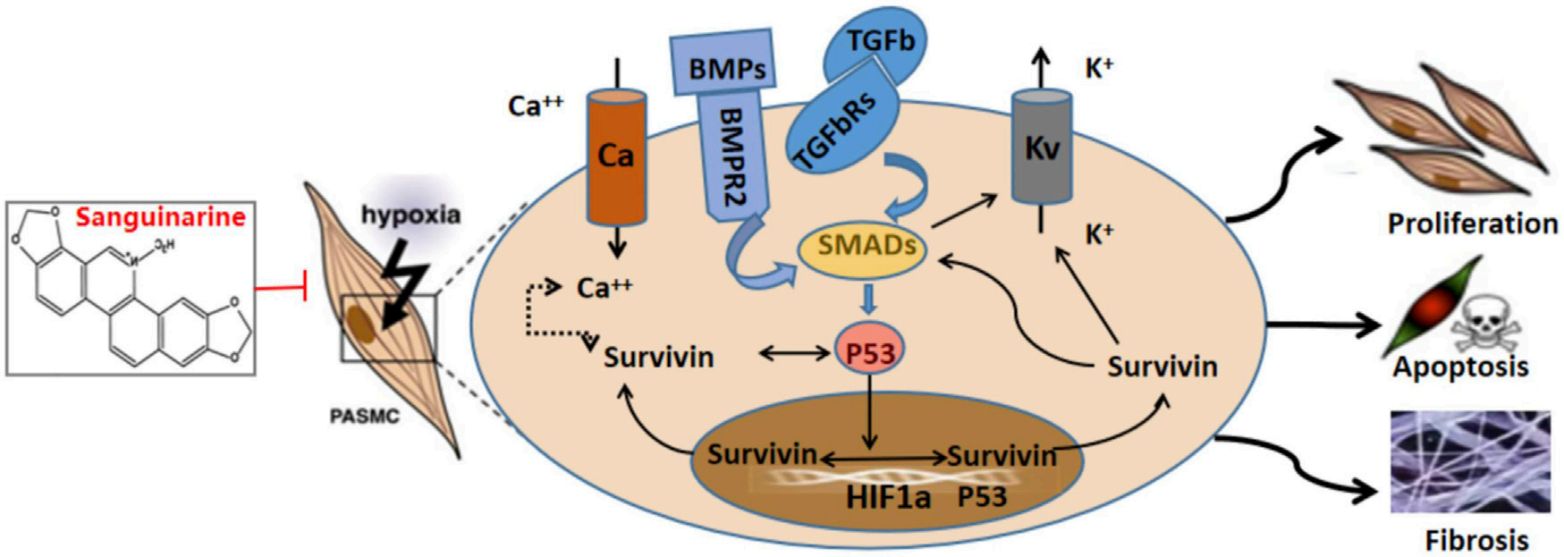
Drawing for effects and the mechanisms of sanguinarine treatment in hypoxia-induced PH.

The occurrence of hypoxic vasoconstriction in the pulmonary circulation relates to the colocalization of an O_2_ sensor and O_2_-sensitive voltage-dependent potassium (Kv) channels in resistance pulmonary arteries. A reduction in Kv channels, in particular Kv1.2, Kv1.5, and Kv2.1, have been proven in the pathogenesis of hypoxia-induced pulmonary hypertension, leading to pulmonary vasoconstriction and vascular remodeling, while the upregulation of Kv channels is of therapeutic significance for pulmonary hypertension (Jackson et al., 2018). In the current study, in hypoxic rats, we observed a reduction in the slope of the current–voltage curve in Kv channels (Figures 8A,B, *p* < 0.05). However, the above reduction of IK was almost recovered completely by treating with sanguinarine (Figures 7A,B, *p* < 0.05), whereas when PASMCs were pre-treated by the Kv channel blocker 4-AP, all above interventions had much less effect on whole-cell voltage K+ current (Figure 7C).

Gene expressions of Kv1.2, Kv1.5, and Kv2.1 were also examined in PAs of experimental animals. The three Kv channels were down-expressed in the hypoxic group (Figures 7D–F, Control group vs. Hypoxia group, P<0.05) but restored again when hypoxic rats were treated with sanguinarine (Figures 7D–F, Sang + Hypoxia group vs. Hypoxia group, *p* < 0.05). These results give another possibility that sanguinarine positively changed PA remolding by directly or indirectly (via survivin, Smad, Ca^++^, etc.) restoring Kv channels.

## Discussion

Pulmonary hypertension (PH) is a fatal disease, classified into five types according to clinical characteristics (as enumerated in the Introduction). However, irrespective of the clinical type, a consistent feature is pulmonary artery remodeling with features similar to those seen in cancer and resulting in increased pulmonary vascular resistance, pulmonary hypertension, right heart failure, and ultimately death. Regarding the mechanism of this cancer-like vascular remodeling, TGF-β/Smad3, BMPR2 pathways, and O_2_-sensitive voltage-gated K + channels are believed to be the important pathways. Previous research has demonstrated that there are cross-talk and interaction between TGF-β/Smad3 and BMP/BMPR2 pathways, and all of them are regulated by apoptotic genes or transcription factors such as survivin,HIF1α, and p53 (Luo et al., 2014; Yang et al., 2020; Young et al., 2006). It has been noted that there is an interactive loop that leads to increased cancer-like stemness, proliferation, metastasis, chemo-resistance, angiogenesis, and immunosuppression, wherein it simultaneously mediates major oncogenic pathways from BMP/Smad to HIF1α and TGFb(Thomas and Wink, 2017). The role of survivin activating TGF-β1/Smad signaling has also been shown in a wide range of cellular processes such as growth, proliferation, differentiation, and apoptosis (Zhao et al., 2020; Park et al., 2019; Liu et al., 2015). We therefore examined these pathways, and the results showed most of them changed in the hypoxia group (Figures 6, 7), consistent to cancer studies and other PH–related research studies (Li et al., 2019b). In the current study, genes and transcription factors related to both cancer and PH including survivin, HIF1α, and p53 were significantly changed in hypoxic rats (Figures 5, 6). These findings confirmed that survivin/HIF1α modulated TGF-β/Smad3 and BMPR2, which is an important mechanism for hypoxia-induced PH as well.

Current treatments for pulmonary hypertension rely on vascular dilatation but do not deal with the underlying pathophysiology, namely pulmonary artery remodeling. The demonstration of the involvement of the survivin/HIF1α and related pathways provides a potential new therapeutic strategy. Sanguinarine (Sang) is a plant alkaloid with the chemical structure of 13-methyl benzodioxole-1,3-dioxolo phenanthradinium. An important intermediate in its synthesis is protopine. Dihydrosanguinarine (DHSA) is formed by hydroxylation of protopine with NADPH, as a reduction cofactor, and molecular oxygen. It has diverse biological activities, including modulation of TGFb and HIF1α. It is also known to induce apoptosis, inflammation, and immunity as reviewed by Mackraj et al. (Cardiovasc Ther. 2008). These effects of sanguinarine were also involved in the development of pulmonary artery remodeling. So, it suggests that sanguinarine would be a potential new therapeutic approach for pulmonary artery remodeling from PH. Importantly, it is enhanced by the previous demonstration in animal and cell studies of a low level of toxicity as well (Wu et al., 2020). In accordance with them, the results from this study illustrated that sanguinarine was remarkably effective in suppressing abnormal proliferation and fibrosis of PASMCs from the exposure to hypoxia (Figures 3, 4). Therefore, sanguinarine significantly improved the PH- and RHF-related characters (elevated PAP, thickness of PA vessels, reduced artery lumen, hypertrophy of RH, etc.), inhibited PASMC proliferation, reduced right heart remolding, and increased the survival rate of rats suffering hypoxia PH. We further investigated the mechanism of anti-vascular remodeling effect of sanguinarine in hypoxic PH. Most of the changes noted previously in the hypoxia group including survivin/HIF1α, TGF-β/Smad3/P53 were restored significantly (Figures 5, 6). The function and expressions of Kv channels were also restored (Figure 7).

Taken together, our findings presented that sanguinarine has a multi-function in suppressing the pathogenesis of PH. Sanguinarine alleviates pulmonary vascular remodeling and fibrosis during hypoxia-induced PH, suppresses the excessive proliferation, and induces apoptosis of PASMCs via inactivation of survivin/HIF1α and their interactions with TGF-β/Smad3/p53. Moreover, hypoxia-induced decrease in function and expression of Kv channels is repressed by sanguinarine, which could be directly or indirectly via survivin/HIF1α, while further study is needed on this (Table 8).

## Conclusion

The study demonstrated that sanguinarine improving the hypoxia-induced characters and pulmonary vascular remodeling *via* survivin/HIF1α and by inhibiting their interactions with BMPR-TGFb/SMAD/p53 signals. It indicated that it is possible that sanguinarine could be a breakthrough anti-vascular remodeling agent for PH, possibly an ideal agent to use in combination with vasodilating agents.

## Data Availability

The original contributions presented in the study are included in the article/Supplementary Material, further inquiries can be directed to the corresponding authors.
